# Function of the Chloroplast Hydrogenase in the Microalga *Chlamydomonas:* The Role of Hydrogenase and State Transitions during Photosynthetic Activation in Anaerobiosis

**DOI:** 10.1371/journal.pone.0064161

**Published:** 2013-05-23

**Authors:** Bart Ghysels, Damien Godaux, René F. Matagne, Pierre Cardol, Fabrice Franck

**Affiliations:** 1 Laboratory of Bioenergetics, Institute of Plant Biology B22, University of Liège, Liège, Belgium; 2 Laboratory of Genetics of Microorganisms, Institute of Plant Biology B22, University of Liège, Liège, Belgium; University of Hyderabad, India

## Abstract

Like a majority of photosynthetic microorganisms, the green unicellular alga *Chlamydomonas reinhardtii* may encounter O_2_ deprived conditions on a regular basis. In response to anaerobiosis or in a respiration defective context, the photosynthetic electron transport chain of *Chlamydomonas* is remodeled by a state transition process to a conformation that favours the photoproduction of ATP at the expense of reductant synthesis. In some unicellular green algae including *Chlamydomonas*, anoxia also triggers the induction of a chloroplast-located, oxygen sensitive hydrogenase, which accepts electrons from reduced ferredoxin to convert protons into molecular hydrogen. Although microalgal hydrogen evolution has received much interest for its biotechnological potential, its physiological role remains unclear. By using specific *Chlamydomonas* mutants, we demonstrate that the state transition ability and the hydrogenase function are both critical for induction of photosynthesis in anoxia. These two processes are thus important for survival of the cells when they are transiently placed in an anaerobic environment.

## Introduction

Despite their ability to produce oxygen by photosynthesis, oxygenic autotrophs can encounter hypoxia or even complete anoxia in a number of situations. This is particularly true during night for microalgae, which may be part of dense O_2_ respiring microbial communities [1]. In response to these situations, a lot of photosynthetic micro-organisms are well adapted to hypoxic or anoxic conditions. For instance, the unicellular green alga *Chlamydomonas reinhardtii*, a resident of soils, shows a complex and versatile fermentative metabolism in which pyruvate, the final product of glycolysis, can be metabolized to lactate or ethanol via the pathways characteristic of higher plants as well as to formate, hydrogen, and acetate by pathways characteristic of facultative anaerobic bacteria [2], [3], [4], [5].

The ability of certain microalgal species to reactivate photosynthesis when transferred from darkness to light in the absence of O_2_ is of major importance for their adaptation to their natural habitat, an aspect which has been poorly considered except in a couple of older works [6], [7]. As a matter of fact, it is well-known that in aerobiosis, the interactions of O_2_ as an electron acceptor with the electron transport chain of plants and microalgae [8] has a strong impact on the induction of photosynthesis during a transition from dark to light [6], [9], [10], [11]. In isolated, anaerobically incubated plant chloroplasts, light fails to induce carbon assimilation unless small amounts of PSI electron acceptors such as O_2_, oxaloacetate or nitrite are added to the preparation [11].

Green algae, which often encounter anaerobic conditions, may therefore rely on particular mechanisms that facilitate the induction of photosynthesis in the absence of oxygen. Several microalgal species, including *Chlamydomonas reinhardtii*, are known for their ability to photo-evolve H_2_ in anaerobiosis as the result of the interaction of ferredoxin with a chloroplast hydrogenase. Electrons activated by the photosynthetic electron transport chain are diverted towards this hydrogenase, which catalyzes the reversible reduction of protons into molecular hydrogen [9]. Due to the extreme oxygen sensitivity of the hydrogenase, H_2_ evolution in the light usually is a transient phenomenon that occurs after pre-incubation of the algae in anoxia. Because of its transient nature H_2_ evolution in some microalgae appears as an irrelevant curiosity of photosynthesis although Melis and co-workers showed that sustainable H_2_ evolution in the light can be achieved in sulphur-deprived algal suspensions [12], [13]. Still, the physiological significance of an oxygen-sensitive hydrogenase linked to the photosynthetic electron transfer chain of microalgae remains puzzling and its selective advantage has not been demonstrated so far.

A possible role of chloroplast hydrogenase in the anaerobic induction of photosynthesis has been suggested by Kessler who observed in the 1970s a link between the occurrence of hydrogenase activity in certain algal species and their ability to activate photosynthesis after dark anaerobic incubation [6]. However, this putative function was never further explored or confirmed. In line with Kessler's results, one may reason that in anaerobiosis, hydrogenase -mediated proton photoreduction could back up for oxygen photoreduction as a transient electron sink and thereby stimulate the activation of photosynthetis during a dark-to-light transition.

Another aspect to take into account is that in anaerobic conditions, the induction of photosynthesis is also influenced by changes in excitation energy distribution between photosystems, known as state transitions. In a rather strict sense, state transitions are defined as redox-regulated changes in the partitioning of light-harvesting pigment-proteins between photosystem II (‘state 1’) and photosystem I (‘state 2’) [14], [15]. In anaerobic *C. reinhardtii* in the dark, non-photochemical reduction of the plastoquinone (PQ) pool triggers a state 1 to state 2 transition. Subsequent illumination leads to a transition back to state 1 along with photosynthetic induction. This return back to state 1 possibly requires the light-triggered re-oxydation of interchain electron carriers and would therefore depend on the presence of PSI electron acceptors, with an inferred role for O_2_ as electron sink by the Mehler reaction [16]. A possible role of the hydrogenase as alternative electron sink in this process has not been suggested so far. In plants the principal role of state transitions is in balancing excitation energy between both photosystems. In the microalga *C. reinhardtii* where the amplitude of this process is much more important than in higher plants, state transitions were shown to have an additional metabolic role [15], [17]. In this microalga state transitions modulate photosynthetic electron transport between linear mode (in state1) and cyclic mode (in state 2) in order to align the NADPH/ATP stoichiometry to the requirements of CO_2_ assimilation and cellular ATP demand [14]. For instance, state transition ability was shown critical for photosynthetic performance of *C. reinhardtii* mutants with respiratory deficiencies [17].

State transitions were never considered as a mechanism that would facilitate the reactivation of photosynthesis of wild-type cells after acclimatization to anoxia in the dark, although this situation is energetically similar to that of dark-adapted mutants deficient for respiration. On the contrary, it is believed that activation of photosynthetic oxygen evolution in anoxic *C. reinhardtii* is delayed as a result of state transitions [16], [18]. This is due to the fact that linear electron transport was shown to be suppressed in the state 2 conformation that prevails under anoxia. The need to return to state 1 was therefore believed to slow down the activation of O_2_ evolution. This was supported by the acceleration of the first detectable O_2_ evolution observed in a mutant locked in state 1 [18].

In the present study we revisited the impact of state transition ability and of hydrogenase activity on the induction of photosynthesis in anaerobic Chlamydomonas. By analyzing activation of photosynthetic electron transport and O_2_ evolution in wild-type and in mutants devoid of hydrogenase activity or locked in state 1, or having both defects, we show that hydrogenase activity and state transition ability are both critical for activation of photosynthesis in the absence of O_2_.

## Results

### Photosynthetic induction of *C. reinhardtii* in anaerobic conditions is facilitated by the presence of hydrogenase as transient electron sink

We started by recording O_2_ evolution and PAM variable fluorescence kinetics of wild-type and hydrogenase-deficient mutant cells (*hydEF* mutation) in the light, following a dark incubation in anaerobic conditions. Variable fluorescence records have the advantage to allow the detection of PSII photochemistry through the proportions of open/closed PSII [19] before the onset of O_2_ evolution, which is strongly delayed in anaerobically incubated algae [6], [10], [16], [20].

**Figure 1:**
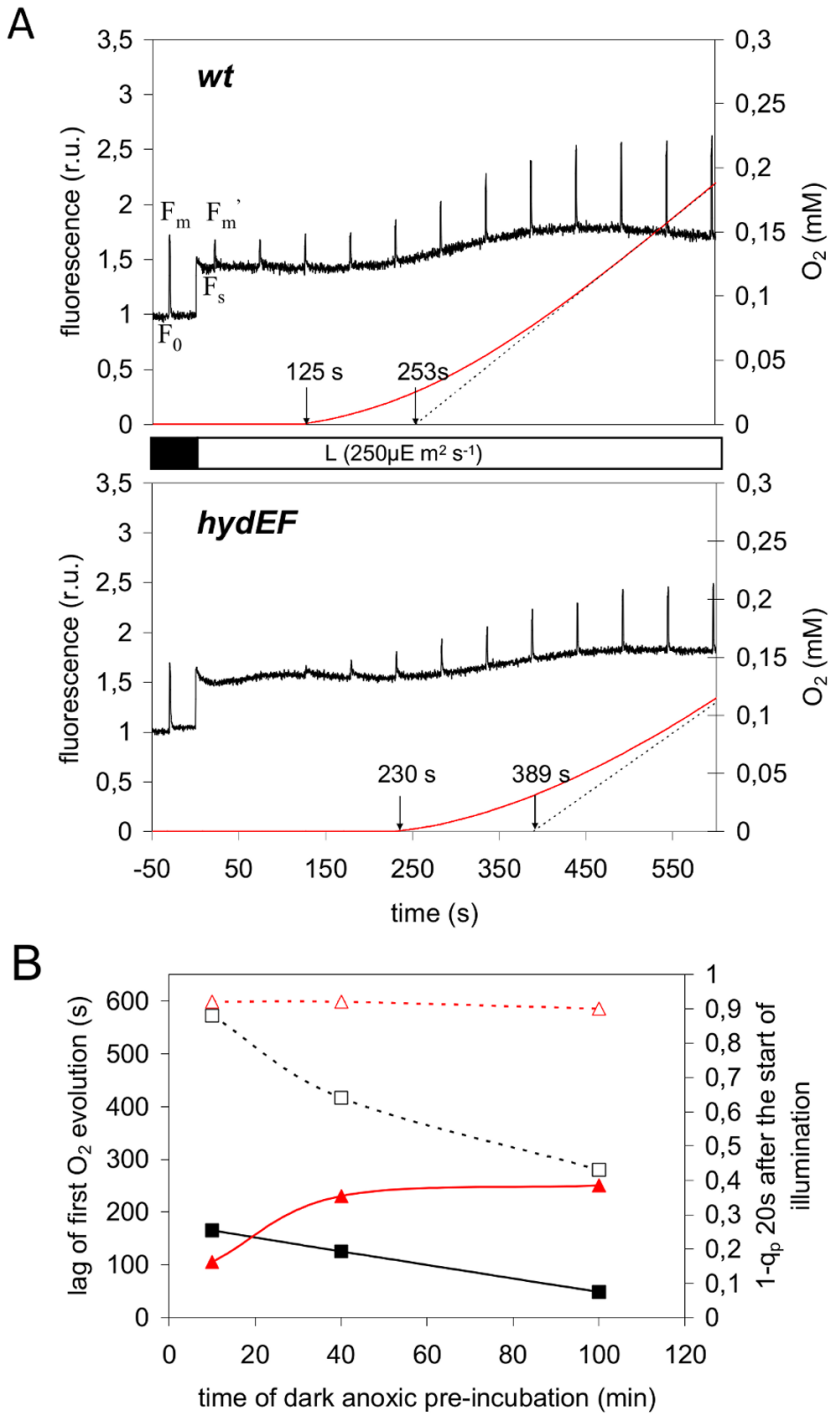
Photosynthesis activation following anoxic incubation in wild-type and hydrogenase deficient *C. reinhardtii*. **A.** Variable (PAM) Chl*a* fluorescence (black) and O_2_ evolution (red) traces upon illumination of *wt* (upper graph) and *hydEF* mutant (lower graph) following a 40 min anoxic incubation in the dark. Saturating light flashes (1 s flashes of 3000 µmol.m^−2^.s^−1^ every 50 s) were applied during the induction process in order to saturate transiently all PSII reaction centers and determine the maximal fluorescence yield Fm′ in the light. Arrows on the time axis indicate the start of detectable oxygen evolution and the intercept of the dashed line drawn from the steady-state O_2_ evolution trace with the time axis. **B.** Proportion of closed PSII centers at time 20 s after the onset of light and duration of the lag of O_2_ evolution in wild-type (squares) and *hydEF* mutant (triangles) as functions of the duration of anoxic acclimatization in the dark. Dashed lines with open symbols: 1-q_p_ parameter calculated from the variable fluorescence traces (see part A) indicating the proportion of closed PSII reaction centers 20 s after the start of illumination. Full lines with closed symbols: time-lag before the first detectable O_2_ evolution.

In typical experiments such as shown in Fig. 1A, induction of photosynthesis in wild-type and *hydEF* mutant cells are compared upon exposure to light (250 µmol.m^−2^.s^−1^) following a 40 min anaerobic incubation in the dark. Clear differences are observed between these two genotypes concerning, on the one hand, the effects of saturating light pulses on the fluorescence yield in the light and, on the other hand, the time at which O_2_ starts to be evolved. In wild-type cells, the increase of the fluorescence yield from a steady-state Fs value to the maximal Fm′ value during the saturating pulses clearly shows that the electron transport chain is not saturated during the 2 min period before the onset of photosynthetic O_2_ evolution. The behaviour of the *hydEF* mutant is quite different since no fluorescence increase is observed upon saturating flashes in that period. This signals the saturation of the electron transport chain in the mutant, likely reflecting a highly reduced state of the pool of PSII electron acceptors as a result of the low rate of their re-oxidation in the light. The wild-type low Fs/Fm′ profile is typical for a more oxidized state of the electron acceptor pools.

These differences in fluorescence signature after the onset of light point to a transient role for the hydrogenase as principal electron sink during the activation of photosynthesis upon anoxic incubation, a condition during which electron sink capacity of the chloroplast is very low. The observed differences between wild-type and *hydEF* fluorescence traces tend to vanish upon continued illumination when the hydrogenase is inactivated by the PSII-driven O_2_ evolution and other electron sinks become available (O_2_, Calvin cycle).

In parallel to the differences in electron transport activity at the onset of illumination we observed a difference in the duration of the lag phase of O_2_ evolution, which is much longer in the hydrogenase deficient mutant (230 s) than in the wild-type (125 s). However, after several minutes of illumination steady-state O_2_ evolution rates are similar. Here also, the convergence of wild-type and *hydEF* traces following continuous illumination illustrates the passing character of the hydrogenase function.

A typical increase of the maximal fluorescence Fm′ upon continuous illumination of the anaerobically incubated algae is also noticed, reflecting the state 2 to state 1 transition linked re-association of LHCII antennae complex to PSII [16]. From these curves no obvious differences in state transition kinetics between wild-type and *hydEF* mutant can be detected.

In a complementary approach we plotted the proportion of closed PSII ([1-q_p_] estimated from the fluorescence traces after 20 s) and the O_2_ lag lengths at the onset of illumination (defined as the time between the start of illumination and the first detectable O_2_ evolution) as function of the length of the dark anaerobic pre-acclimatization (Fig. 1B). Previous work has shown that active hydrogenase accumulation is progressive in anaerobic Chlamydomonas and reaches a maximum only after 150 min anaerobic incubation [21]. After a short anaerobic acclimatization (10 min) wild-type and *hydEF* behave similarly: the proportion of closed PSII in the light (1-q_p_) was high in both cases, revealing a transient congestion of PSII-driven electron transport at the onset of illumination, due to a lack of electron acceptors, which was also reflected by the long lag phases of O_2_ evolution. In the wild-type but not in the *hydEF* mutant, 1-q_p_ values decreased with increasing duration (40 min, 100 min) of the dark anaerobic pre-acclimatization. This anaerobic acclimatization effect can be ascribed to the progressive increase of hydrogenase activity [21], [22]. In parallel to the drop of 1-q_p_ a decrease in the lag length for O_2_ evolution is observed in wild-type. An opposite evolution is found for the *hydEF* mutant, which exhibited an increase of the O_2_ lag duration with longer anaerobic pre-incubation periods while the proportion of closed PSII (1-q_p_) remained high (see also Fig. S1). These data confirm the link between PSII-driven electron transport and hydrogenase activity during the early stages of photosynthesis activation in anaerobiosis.

**Figure 2:**
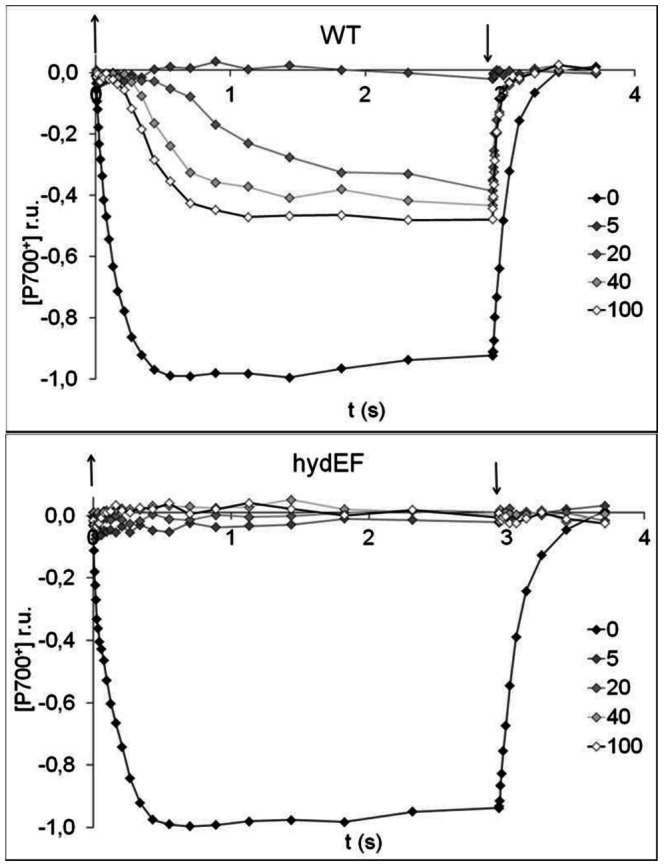
Kinetics of P700 photo-oxidation in function of anoxic acclimatization length in wild-type Chlamydomonas and in a hydrogenase deficient mutant. Kinetics of P700 oxidation following illumination (300 µmol.m^2^.s^−1^) in the presence of DCMU (10 µM) after increasing times (5,20,40,60 and 100 min) of dark anaerobic acclimatization in wild-type and *hydEF* mutant. Oxygen was removed by addition of glucose oxidase and β-D-glucose. P700^+^ accumulation in the light was monitored as the decrease of transmission at 705 nm. The start of illumination is indicated by a downward arrow. An upward arrow indicates the end of the illumination. The depicted traces are corrected for contributions caused by redox variations of the plastocyanine pool (740 nm transmission signal) and normalized to the steady-state level of oxidized P700, reached during illumination of the air-exposed sample in presence of DCMU (0 min trace). Hence [P700^+^ ] is expressed in relative units comprised between 0 (in the dark) and 1 (light steady-state oxidation level, air exposed).

Since the hydrogenase operates as an alternative electron sink for photosynthetic electron transport by accepting electrons from reduced Fd downstream of PSI, we decided to assess the correlation between hydrogenase activity and electron sink capacity downstream of PSI by recording kinetics of the light-induced oxidation of the primary PSI donor P700. Since the P700 oxidized form has lower absorbance at 705 nm than its reduced form, P700 oxidation in the light can be followed using a Joliot type spectrometer (Fig. 2). Measurements were done in the presence of the PSII inhibitor DCMU to avoid fast P700^+^ re-reduction in the light. Very slow or no P700 oxidation in the light was observed after a short (5 min) anoxic incubation of the wild-type or *hydEF* mutant, revealing the lack of electron acceptors downstream of PSI after short exposure to anoxia [23]. In this condition, there is little to no hydrogenase activity in wild-type and thus wild-type and *hydEF* mutant both show very slow P700 oxidation kinetics. Just like for the PSII fluorescence derived parameters, a strong effect of the anaerobic acclimatization length is observed for the wild-type but not for the *hydEF* mutant on P700 photo-oxidation kinetics, an effect we assign to the progressive induction of hydrogenase in wild-type only. After 40 to 100 min anaerobic incubation wild-type cells have reached maximal P700 photo-oxidation capacit, that corresponded to about 50% of that in aerobiosis.

### Lack of hydrogenase activity in a state 1 locked mutant is detrimental to its ability to induce photosynthesis in an anaerobic environment

It was previously proposed that the start of linear electron flow and concomitant O_2_ evolution in anaerobically adapted cells having adopted the state 2 conformation, would require a (partial) return to state 1 [16], [18], [24]. The slow induction of the O_2_ evolution in anaerobic conditions would hence be explained by the relatively slow rate of the state transition process. Our present observations show that the activation of linear photosynthetic electron transport and O_2_ evolution in anoxic Chlamydomonas cells is also influenced by the electron sink capacity in the chloroplast, independently of the state transition process. In anaerobiosis, the rate of photosynthesis induction would thus be influenced by two parameters, the state transition process and the electron sink capacity associated to the activity of the hydrogenase.

**Figure 3:**
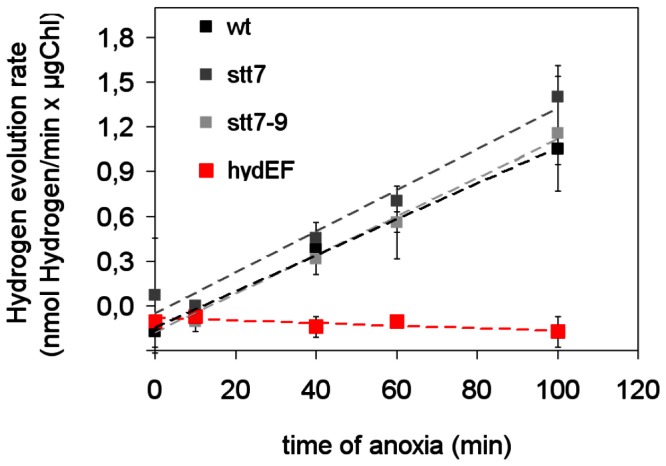
Increasing hydrogen evolution ability with progressing anaerobic incubation length in wild-type and in the state transition mutants *stt7* and *stt7-9*. Initial rates of hydrogen evolution in the light (300 µmol.m^2^.s^−1^) in function of the anoxic acclimatization length of the algal suspensions. Data are expressed as the average of three independent experiments. Error bars represent ±SD.

To better determine the contribution each of these two parameters individually, we used the *stt7* mutant, locked in state 1 [25]. We first analyzed the hydrogen evolution ability of this mutant (and of the *stt7-9* mutant, a clone allelic to *stt7*). As shown in Fig. 3, H_2_ photoevolution rates increased in similar ways in *stt* mutants or in wild-type with incubation times under anoxia. As expected, the *hydEF* mutant did not shown H_2_ photoevolution.

**Figure 4:**
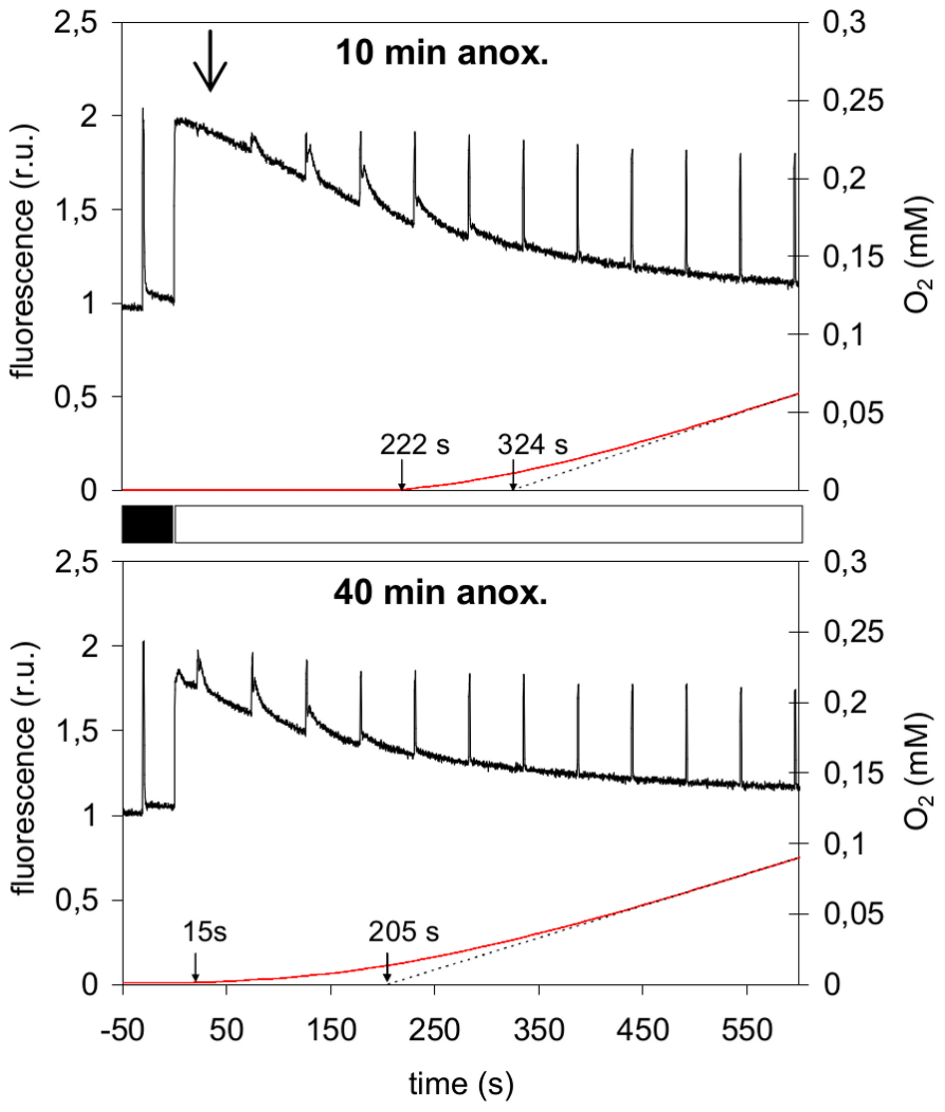
Photosynthesis activation following anoxic incubation in a state transition disabled mutant. Chl*a* fluorescence (black) and O_2_ evolution (red) traces upon illumination (250 µmol.m^−2^.s^−1^) of *stt7*, a mutant locked in state 1, following either a 10 min (upper graph) or 40 min (lower graph) anoxic incubation in the dark . Saturating light flashes (1 s flashes of 3000 µmol.m^−2^.s^−1^ every 50 s) were applied during the induction process in order to saturate transiently all PSII reaction centers and determine the maximal fluorescence yield Fm′ in the light. Arrows on the time axis indicate the start of detectable oxygen evolution and the intercept of the dashed line drawn from the steady-state O_2_ evolution trace with the time axis. The large arrow in the upper graph marks the moment of the first saturating light flash.

We then compared the photosynthetic activation kinetics in the *stt7* mutant and in the wild-type strain after different incubation times under anoxia. Incubation times of 40 min, or longer, resulted in shorter O_2_ evolution lags in the *stt7* mutant (15 s, Fig. 4B) than in the wild-type (125 s, Fig. 1A,B), which seems to confirm the statements made in previous studies [16], [18], [24] that establishment of state 2 is detrimental to photosynthetic activation in anoxia. However this picture could easily be reversed by cutting down the anaerobic acclimatization time as shown in Fig. 3A. Following a 10 min anoxic acclimatization, the lag of O_2_ evolution in *stt7* (222 s, Fig. 4A) is indeed longer than in the wild-type strain (180 s, Fig. 1B). The variable fluorescence shows that the rate of PSII photochemistry shortly (20 s) after the onset of illumination is severely obstructed when *stt7* cells are incubated for 10 min in anaerobic conditions (Fig. 4A) but restored after a 40 min incubation period in anaerobiosis (Fig. 4B).

The strong dependence of the activation of photosynthesis on hydrogenase activity in the *stt7* mutant thus shows that the induction of photosynthetic O_2_ evolution in anoxia is basically limited by electron acceptor capacity. Only when sufficient electron acceptor capacity is insured by the hydrogenase activity, the slow rate of the reverse state transition can become a limiting factor for the induction of linear electron transport and O_2_ evolution as shown in previous studies [16], [18], [24].

For assessing the relative impact of hydrogenase activity and state transition on the process of photosynthesis induction in anoxia, we used a genetic approach and constructed a double mutant both locked in state 1 and deficient in hydrogenase activity. We mated *stt7-9 mt+* (17) with *hydEF mt-* cells and the progeny clones were analyzed by fluorescence. First, Fv/Fm values were measured in atmospheric conditions. The plates were then placed in an anoxic atmosphere for 4 hours after which Fv/Fm values were measured again in order to distinguish clones that had passed to state 2 (lower Fv/Fm values) from those who stayed in state 1 (unchanged Fv/Fm values). Subsequently, the variable fluorescence of the clones upon actinic illumination in this anoxic atmosphere was analyzed in order to identify the hydrogenase deficient clones (by lack of flash-induced variable fluorescence in the light). From 18 clones analyzed, two clones displaying a double mutant phenotype were identified and one of them was used for deeper analysis. PCR analysis confirmed the presence of the mutation in the *hydEF* gene in this double mutant (Fig. S2), which led to its inability to photoevolve H_2_ under anoxia (Fig. S3). Absence of state transition under anoxia was also verified in the double mutant (Fig. S4).

**Figure 5:**
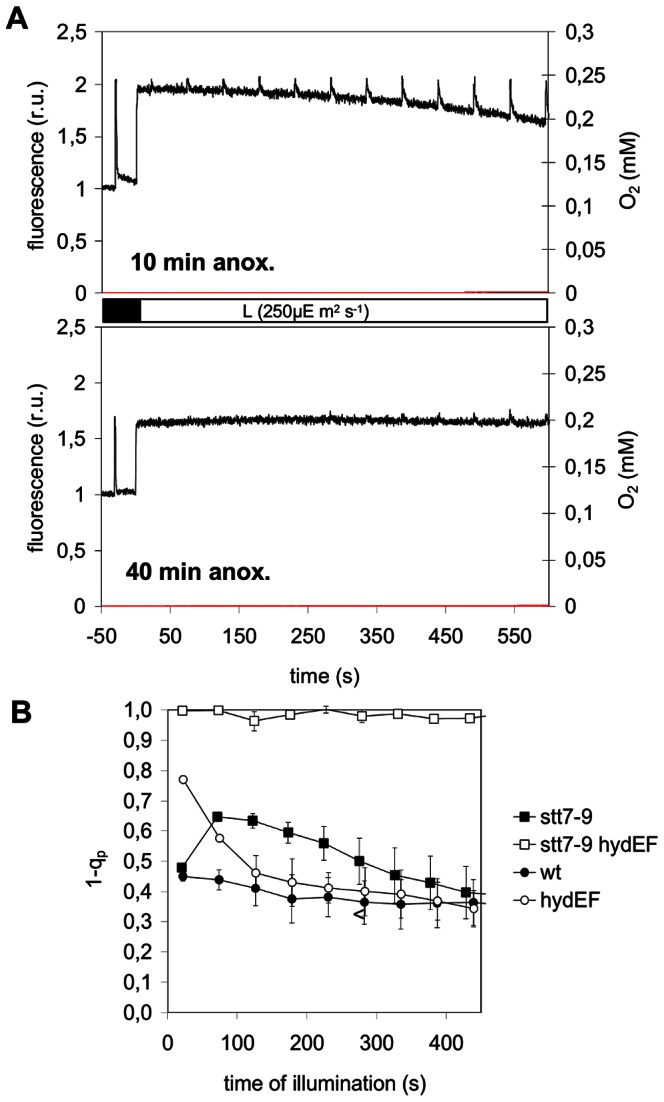
Photosynthesis activation following anoxic incubation in a double mutant deficient for both state transition and hydrogenase activity. **A.** Chl*a* fluorescence (black) and O_2_ evolution (red) traces upon illumination (250 µmol.m^−2^.s^−1^) of a double mutant *stt7*-9/*hydEF*, a hydrogenase deficient mutant locked in state 1, following a 10 min (upper graph) or 40 min (lower graph) anoxic incubation in the dark . Saturating light flashes (1 s flashes of 3000 µmol.m^−2^.s^−1^ every 50 s) were applied during the induction process in order to saturate transiently all PSII reaction centers and determine the maximal fluorescence yield Fm′ in the light. **B.** Evolution of the fraction of closed PSII centers (1-qp), in wild-type and in *hydEF*, *stt7-9* and *stt7-9hydEF* mutant suspensions of algae during illumination (250 µmol.m^−2^.s^−1^) following a 40 min anoxic incubation in the dark. Error bars indicate standard error of the mean of three independent experiments.

We analyzed the ability of the double mutant to induce photosynthesis after anaerobic adaptation. Fig. 5, A and B show that in the double mutant the ability to activate photosynthesis in anoxic conditions is severely compromised, especially after longer anaerobic acclimatization times. After 40 min acclimatization to anoxia in the dark (Fig. 5A, lower trace), the electron transport chain remained saturated in the light (almost no flash-induced fluorescence increase in the light) and O_2_ was not evolved within a 10 min time-scale, whereas single mutants (*hydEF* or *stt7*) both had reached steady-state O_2_ evolution in this time-scale (see Fig. 1A and Fig. 5B). The extreme phenotype of the double mutant can be appreciated on a quantitative basis by comparing the kinetics of the parameter 1-q_p_ (the proportion of closed PSII) during illumination in single mutants and in wild-type (Fig. 5B). Only in the double mutant this proportion remained close to 1.0 when in single mutants or in wild-type it reached values around 0.4 after transient changes during photosynthetic activation.

The absence of photosynthetic activation in the double mutant shows that, in a hydrogenase deficient background, Chlamydomonas resorts to the ability to adopt a state 2 conformation in order to activate photosynthesis starting from anoxia, revealing a so far unknown function of state transitions in Chlamydomonas.

## Discussion

The induction of a chloroplast Fe-Fe hydrogenase, mediating the light-driven reduction of H^+^ to H_2_ in the chloroplast stroma, is one of the most enigmatic characteristics of the anaerobic response of *C. reinhardtii*. The high metabolic cost of hydrogenase maturation, requiring ATP and a set of accessory proteins, is paradoxical to the transient, almost anecdotic, character of hydrogen photo-evolution. In this study we resorted to a genetic and functional approach in order to show that micro-algal hydrogen photo-evolution ability can become critical for *C. reinhardtii* to restore photosynthetic activity under anoxic conditions.

We first showed that hydrogenase activity, despite of its transient nature, has a positive impact by shortening the lag phase of re-activation of oxygenic photosynthesis following an anoxic incubation in the dark. By comparison of variable fluorescence and P700 oxidation kinetics between a hydrogenase deficient strain and a wild-type strain we showed that *C. reinhardtii* resorts to the hydrogenase as alternative electron sink, allowing linear electron flow during dark-light transitions in anoxic conditions prior to the activation of CO_2_ assimilation. In the absence of O_2_, the hydrogenase obviously replaces the Mehler reaction that fulfils a similar role in atmospheric conditions [26]. In general terms, the lag of CO_2_ assimilation at the onset of an illumination can be ascribed to the inactive state of Calvin cycle enzymes in the dark and the need for subsequent activation by pH and redox (Fd- thioredoxin) dependent activation. In anaerobiosis, the start of CO_2_ assimilation is additionally compromised by the distortion of the energetic and redox balance of the chloroplast stroma, a consequence of the absence of oxidative phosphorylation in the mitochondria. In such conditions electron flow between water and NADP is impaired by the highly reduced state of stromal NADP(H) pools while depleted ATP pools interfere with the Calvin cycle dependent regeneration of oxidized stromal equivalents. Dissipation of electrons through the hydrogenase valve allows for linear electron flow to take place without NADP reduction but with the associated formation of an electrochemical gradient that drives ADP phosphorylation. Hence, we suggest that the hydrogenase contributes to restore the ATP to NADPH ratio suitable for the start of CO_2_ assimilation in anaerobically adapted algae.

A complementary role of the hydrogenase during anoxic dark-light transitions could also be to accelerate the light-induced increase in stromal pH that triggers activation of CO_2_ fixing reactions [27]. It is known for instance that Rubisco has a pH optimum of around 8.8 but is relatively inactive at pH 7.0 [28]. Moreover, the charge deficit caused by stroma alkalinisation is compensated by import of Mg^2+^, co-factor of Rubisco [29]. Interestingly, the hydrogenase contributes twice to the alkalinisation of the stroma, indirectly by allowing electron transport coupled proton gradient formation in highly reducing conditions and directly by consuming stromal protons for H_2_ formation.

Despite these effects, absence of hydrogenase activity merely extends the activation time in anaerobiosis but does not prevent it. This lack of a stronger phenotype can better be understood if we take into consideration the previously demonstrated flexibility of the Chlamydomonas chloroplast in dealing with similar situations of high metabolic redox pressure and ATP deficiency by the mechanism of state transitions. This was recently exemplified in mutants with defects in mitochondrial respiration [17]. State transitions were indeed suggested to interfere with the induction of photosynthesis in anaerobic algal cells. However previous studies suggested that they would essentially slow down the activation of O_2_ evolution by the algae [16]. This was assigned to conformational constraints of the photosynthetic apparatus in state 2 cells, which impair linear electron flow and allow almost exclusively cyclic electron transport [18], [24]. Consequently, the activation of photosynthetic O_2_ evolution in anoxic conditions would be slow due to the dependence of linear electron flow on a return to state 1.

However, the present observations lead to another contrasting vision according to which the initial state 2 configuration promotes the activation of photosynthesis in anoxia by favouring cyclic photophosphorylation. It was shown before that through the mechanism of state transitions the chloroplast can adapt to function in conditions of impaired mitochondrial phosphorylating capacity [17], [30]. When the depletion of the cellular ATP reserves and the following accumulation of reducing equivalents in the chloroplast triggers a transition to state 2, the consequent re-shuffling of photosystems, electron transporters and light harvesting antennae favor cyclic photo-phosphorylation [24], [31], [32] which allows the generation of ΔpH without the net production of stromal reducing equivalents. In this work, and in line with previous studies [33], an increased rate of cyclic electron flow under anoxia was found for the wild-type compared to state transition mutants. This was assessed here by measuring rates of P700^+^ re-reduction after short illumination in presence of DCMU (28±4 s^−1^ in wild-type and 18±3 s^−1^ in the *stt7-9* mutant (data not shown)). High rates of cyclic electron transport in state 2 likely help to restore the required ATP/NADPH stoichiometry for CO_2_ fixation. The importance of state transitions for photosynthetic efficiency in an ATP depleted metabolic context was illustrated earlier by the strongly affected photo-autotrophic growth of mutants combining a mitochondrial respiration defect with state transition impairment [17]. In dark anoxic conditions, impairment of mitochondrial respiration similarly results in ATP depletion in the chloroplast, which interferes with CO_2_ assimilation at the beginning of illumination. Owing to the state 2 conformation, cyclic phosphorylation can bridge the ATP deficit that hampers activation of the Calvin cycle.

In the present work, we managed to reconcile these two seemingly conflicting viewpoints concerning the role of state transitions by considering the influence of the hydrogenase, a factor which was ignored in previous works. We show that in a hydrogenase deficient context Chlamydomonas ability to perform state transitions becomes critical for its capacity to resume photosynthesis in anaerobic situations. Deprived of electron sinks in the absence of hydrogenase, Chlamydomonas cells fall back on the state 2 conformation to restore stromal ATP levels and consequently enable the activation of CO_2_ reduction and liberation of electron acceptor pools downstream of PSI. However, as soon as the genetic background and the length of anaerobic incubation allow for the accumulation of active hydrogenase, photosynthesis induction is released from its limitation by electron sink capacity. In that situation, O_2_ evolution will resume sooner in a mutant locked in a state 1 conformation favorable to linear electron transport, than in a wild-type strain which is in state 2. This was also found in the present study and places our report in line with the observations and statements by others [16], [18], [24].

In conclusion, we here show that state transitions and the induction of a chloroplast hydrogenase, independently promote the activation of photosynthesis in anoxic conditions. Although none of these mechanisms appears to be critical when knocked-out individually, a combination of both defects strongly compromises the ability of *C. reinhardtii* to resume photosynthesis in anoxic situations. For a soil resident alga like Chlamydomonas surrounded by dense respiring microbial populations, O_2_ deprivation is a frequently returning stress. We may therefore assume that the extensive state transition capacity of this alga together with its ability to dissipate electrons via hydrogenase-mediated H_2_ photo-evolution reflect the strong ability of Chlamydomonas cells to switch metabolism between anaerobic periods in the dark and aerobic periods in the light.

## Materials and Methods

### Strains and Growth Conditions

The *Chlamydomonas* wild-type strain 137AH is derived from the 137c strain (cc-1373 of the Duke University). The *hydEF* mutant [34] is a gift from M. Seibert. *Stt7–9* is a clone allelic to *stt7* (17), which can be crossed easily, unlike the original strain (gift from J.-D. Rochaix). The double mutants *hydEF stt7–9* were obtained by crossing the *hydEF mt^−^* mutant with a *stt7–9 mt* ^+^ mutant using standard procedures. We isolated the double mutants by using their fluorescence characteristics under an anoxic atmosphere, created by placing the petri dishes with progeny colonies into air-tight plastic bags (GasPak™ EA Pouch System, BD) in the dark. Cells were cultivated at 50 µmol photons m^−2^s^−1^ in mixotrophic (TAP) conditions.

### PCR analysis

PCR fragments were amplified from total DNA as in [35].

### Oxygen Evolution and variable Chla fluorescence analysis

Oxygen evolution and the PSII photochemical activity in the light was measured simultaneously using a Clark electrode connected to a modulated fluorometer (type MFMS, Hansatech Instruments) in TAP liquid medium supplemented with 10 mM NaHCO_3_. q_p_ was calculated as (Fm′-Fs)/(Fm′-F_0_), where Fm′ is the maximum fluorescence emission level induced by a pulse of saturating light (≈5,000 µmol.m^−2^.s^−1^), Fs the fluorescence emission level during continuous actinic illumination and F_0_ the fluorescence emission level prior to the dark-light switch [19]. Chlorophyll concentration was adjusted to 15 µg.ml^−1^ by diluting the cells in culture medium collected by centrifugation (in order to avoid changing medium conditions prior to the experiment).

### Spectroscopic measurements of P700 oxidation

Cells were harvested during exponential growth (2*10^6^ cells.ml^−1^) and were resuspended at a chlorophyll concentration of 15 µg.ml^−1^ in TAP medium with the addition of 15% (w/v) Ficoll to prevent cell sedimentation. In vivo P_700_ oxidation kinetics were measured in the presence of 3-(3′,4′-dichlorophenyl)-1,1-dimethylurea (DCMU), at room temperature using a JTS spectrophotometer (Biologic, France). Continuous light was provided by a red source (630 nm), which was switched off transiently while measuring light transmission at 705 nm. The traces were corrected for contributions caused by redox variations of the plastocyanine pool (740 nm transmission signal).

### Hydrogen evolution

Hydrogen evolution was measured using an oxygen-sensitive Clark electrode (Oxygraph, Hansatech Instruments) modified to only detect hydrogen (Oxy-Ecu, Hansatech Instruments). Near-saturating actinic light was provided by a home-made light system composed of white and green LED's. The entire set-up was placed in a plastic tent under anoxic atmosphere (N_2_) to avoid contamination of anaerobic samples by oxygen while filling the measuring cell.

### 77K fluorescence emission

Fluorescence emission spectra at 77 K were recorded using a LS 50B spectrofluorometer (Perkin Elmer). The excitation wavelength was 440 nm. Excitation and emission slits were 10 and 5 nm, respectively. A broad blue filter (CS-4-96, Corning, NY) was placed between the excitation window and the sample to minimize stray light. Cells were treated to induce state transitions before freezing in liquid nitrogen. Chl concentration was lower than 2 µg ml^−1^, and it was verified that no changes in the intensity ratio of the 685- and 715-nm emission bands arose from re-absorption artifacts. Spectra were corrected for the wavelength-dependent photomultiplier response.

## Supporting Information

Figure S1 **A.** Influence of the duration of anoxic acclimatization on the lag phase of O_2_ evolution following illumination (250 µmol.m^−2^.s^−1^) in WT, *hydEF* and *stt7* mutant cells. The bars show the average difference in lag phase duration between 40 min and 10 minutes acclimatized cells, which were taken from a single algal culture. The lag phases of WT cells and *stt7* placed 40 minutes in anoxia are systematically shorter than those of cells placed only 10 minutes in anoxia, leading to a negative difference in the duration of the lag phase between the 40 min and 10 min condition. This is not the case for the hydrogenase deficient mutant *HydEF*, for which we systematically observed an increase of the lag phase following a longer incubation in anoxia. Data are expressed as the average of five (WT) or three (*HydEF* and *stt7*) independent experiments. Error bars represent ±SD. **B.** Influence of the duration of anoxic acclimatization on the fraction of closed PSII centers (1-qp) measured shortly (20 s) after the start of illumination (250 µmol.m^−2^.s^−1^) in WT, *hydEF* and *stt7* mutant cells. 10 minutes anoxic acclimatized cells are compared to 40 min anoxic acclimatized cells. The 1-qp parameter was derived from individual variable fluorescence traces such as in Fig. 1A, recorded after 10 minutes or 40 minutes anoxic incubation of the cells. Data are expressed as the average of five (WT) or three (*HydEF* and *stt7*) independent experiments. Error bars represent ±SD.
(TIFF)

Figure S2 Analysis by 77 K fluorescence spectroscopy of state transition ability of the different strains used in this study. Two different pretreatments were applied to cell suspensions before freezing in liquid N_2_: black traces: dark incubation with vigorous shaking to insure strong oxygenation of the cultures; red traces: anoxic incubation in the dark for 15 min upon removal of O_2_ by addition of glucose oxidase and β-D-glucose. A, Fluorescence emission spectra (77 K) of the wild-type control (dashed lines) and the *stt7-9* mutant (full lines). B, Fluorescence emission spectra (77 K) of the *hydEF* mutant (dashed lines) and the *stt7-9hydEF* double mutant (full lines).
(TIFF)

Figure S3 Hydrogen evolution ability of WT and *hydEF* and *stt7-9hydEF* mutant strains. Hydrogen evolution ability in the light (300 µmol photons m^2^ s^−1^) measured by polarography after 100 min anoxic acclimatization of the algal suspension (following oxygen depletion by glucose oxidase and β-D-glucose addition.)
(TIFF)

Figure S4 PCR analysis for detecting the presence of an interrupted *HydEF* gene in the single *hydEF* mutant (*arg7* insertion between the 8^th^ and 9^th^ exon as indicated in Posewitz *et al.*, 2004 [34] and *stt7-9hydEF* double mutant. Fw (Position Fw primer: 502618) and Rv Primers (Position Rv primer: 503745) were chosen at either side of the presumed insertion site in order to yield 1100 bp fragment in WT. The relatively short elongation step in the thermal cycling program did not allow the amplification of the much longer genomic fragment including also the *arg7* insert, neither in the *hydEF* nor in the *stt7-9hydEF* mutant strains. The amplification of an independent genomic fragment of similar size order (600 bp) was used as a positive control for DNA quality and reaction conditions.
(TIFF)
