# Supplementary figures and images for: Function of the Chloroplast Hydrogenase in the Microalga Chlamydomonas: The Role of Hydrogenase and State Transitions during Photosynthetic Activation in Anaerobiosis
Source: PLoS One. 2013 May 23;8(5):e64161. doi: 10.1371/journal.pone.0064161 (PMC3662714; doi:10.1371/journal.pone.0064161)

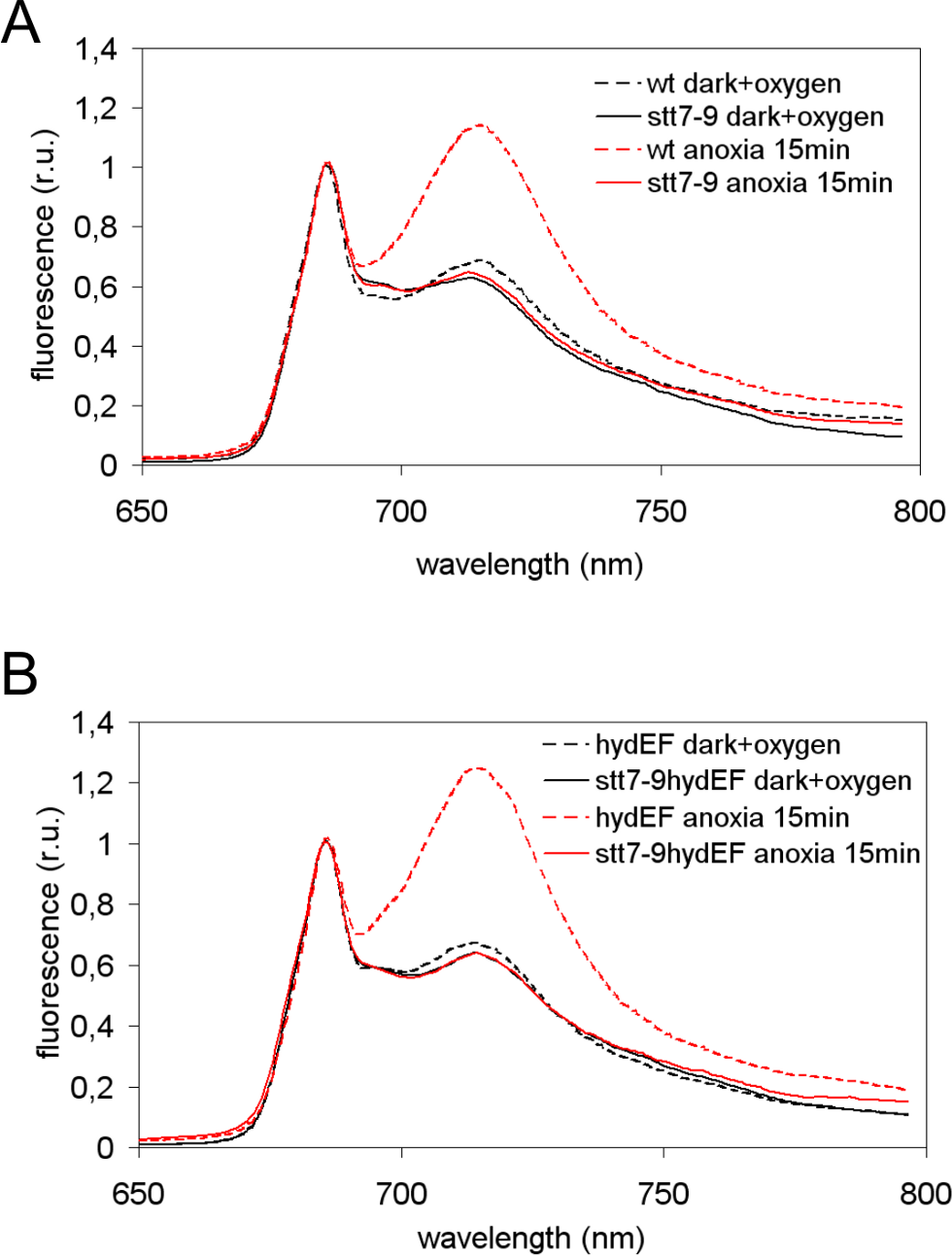

Supplement: Figure S2 — Analysis by 77 K fluorescence spectroscopy of state transition ability of the different strains used in this study. Two different pretreatments were applied to cell suspensions before freezing in liquid N2: black traces: dark incubation with vigorous shaking to insure strong oxygenation of the cultures; red traces: anoxic incubation in the dark for 15 min upon removal of O2 by addition of glucose oxidase and β-D-glucose. A, Fluorescence emission spectra (77 K) of the wild-type control (dashed lines) and the stt7-9 mutant (full lines). B, Fluorescence emission spectra (77 K) of the hydEF mutant (dashed lines) and the stt7-9hydEF double mutant (full lines). (TIFF) [file pone.0064161.s002.tiff]

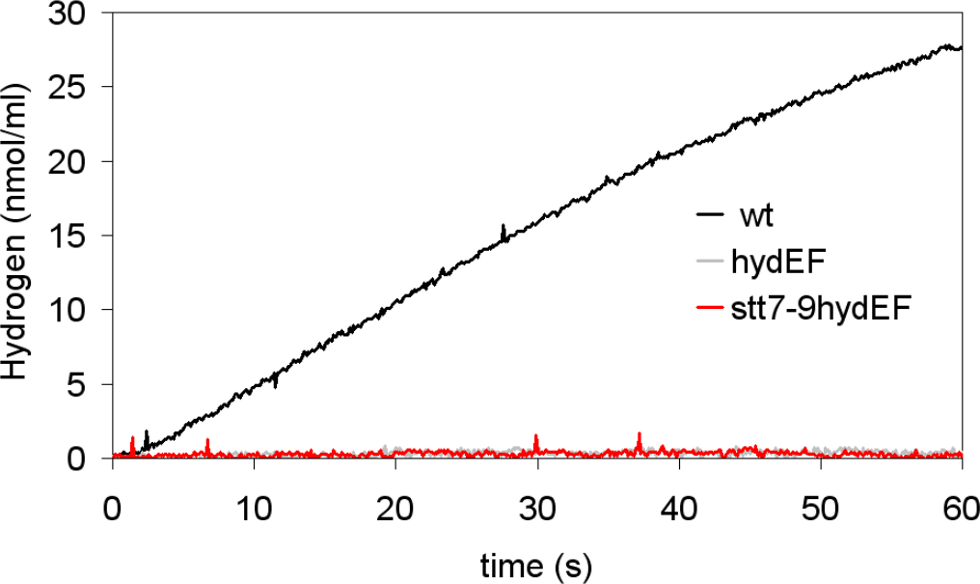

Supplement: Figure S3 — Hydrogen evolution ability of WT and hydEF and stt7-9hydEF mutant strains. Hydrogen evolution ability in the light (300 µmol photons m2 s−1) measured by polarography after 100 min anoxic acclimatization of the algal suspension (following oxygen depletion by glucose oxidase and β-D-glucose addition.) (TIFF) [file pone.0064161.s003.tiff]
